# Supplementary material for: An Integrative Bioinformatic Analysis for Keratinase Detection in Marine-Derived Streptomyces
Source: Mar Drugs. 2021 May 21;19(6):286. doi: 10.3390/md19060286 (PMC8224001; doi:10.3390/md19060286)
Supplement: Supplementary file 1 [file marinedrugs-19-00286-s001.zip › marinedrugs-1203633-SI/Supplementary_files5.13/Fig_S2.pdf]

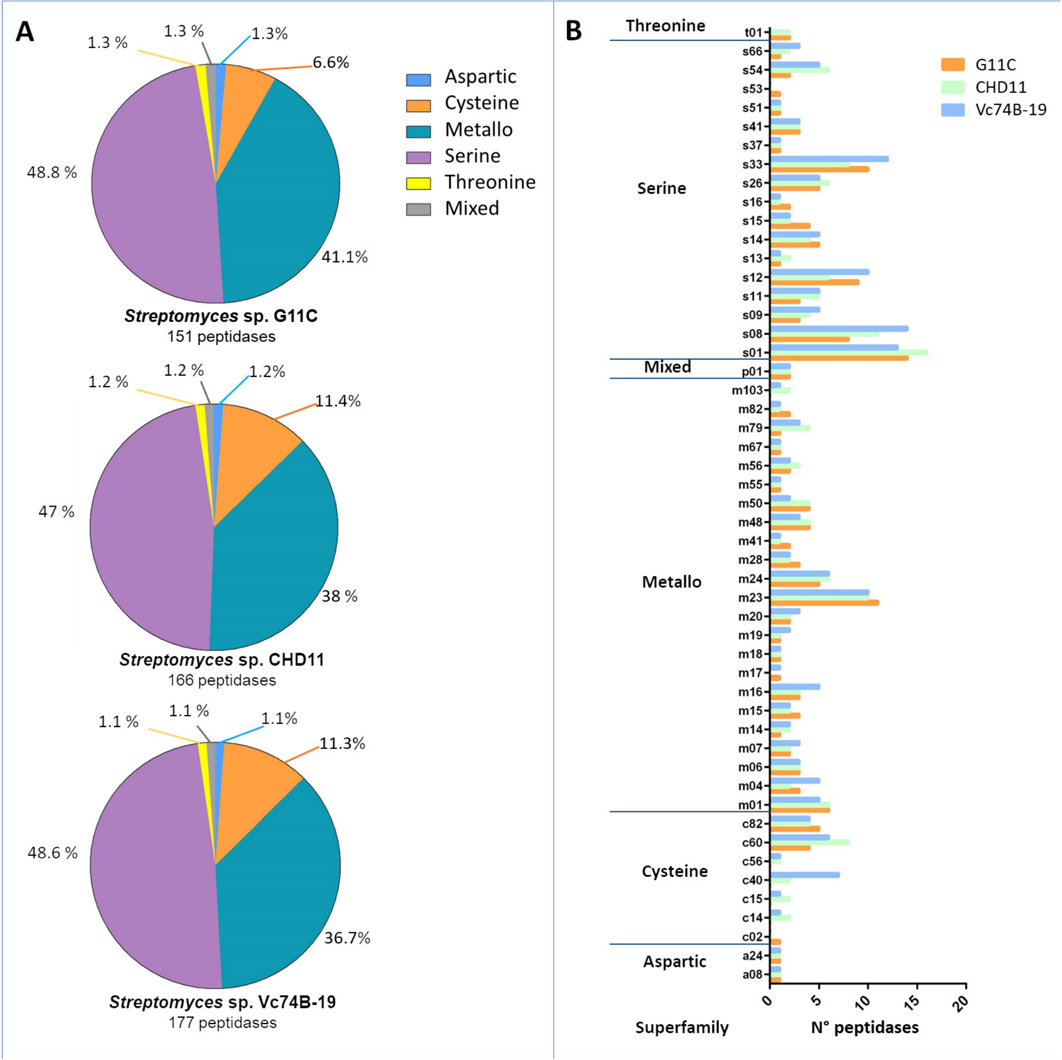
A
1.3 %
1.3 %
1.3%
6.6%
48.8 %
41.1%
Streptomyces sp. G11C
151 peptidases
1.2 %
1.2 %
1.2%
11.4%
47 %
38 %
Streptomyces sp. CHD11
166 peptidases
1.1 %
1.1 %
1.1%
11.3%
48.6 %
36.7%
Streptomyces sp. Vc74B-19
177 peptidases
Aspartic
Cysteine
Metallo
Serine
Threonine
Mixed
B
Threonine
Serine
Mixed
Metallo
Cysteine
Aspartic
t01
s66
s54
s53
s51
s41
s37
s33
s26
s16
s15
s14
s13
s12
s11
s09
s08
s01
p01
m103
m82
m79
m67
m56
m55
m50
m48
m41
m28
m24
m23
m20
m19
m18
m17
m16
m15
m14
m07
m06
m04
m01
c82
c60
c56
c40
c15
c14
c02
a24
a08
G11C
CHD11
Vc74B-19
0
5
10
15
20
Superfamily
N° peptidases
