# Supplementary material for: An Integrative Bioinformatic Analysis for Keratinase Detection in Marine-Derived Streptomyces
Source: Mar Drugs. 2021 May 21;19(6):286. doi: 10.3390/md19060286 (PMC8224001; doi:10.3390/md19060286)
Supplement: Supplementary file 1 [file marinedrugs-19-00286-s001.zip › marinedrugs-1203633-SI/Supplementary_files5.13/Sup_Figures_Valencia2021_Rev.pdf]

marine drugs

MDPI

Article

An Integrative Bioinformatic Analysis for Keratinase Detection in Marine-derived *Streptomyces*

Ricardo Valencia [1†], Valentina González [1†], Agustina Undabarrena [1], Leonardo Zamora-Leiva [1], Juan A. Ugalde [2] and Beatriz Cámara [1,*]

Supplemental Figures

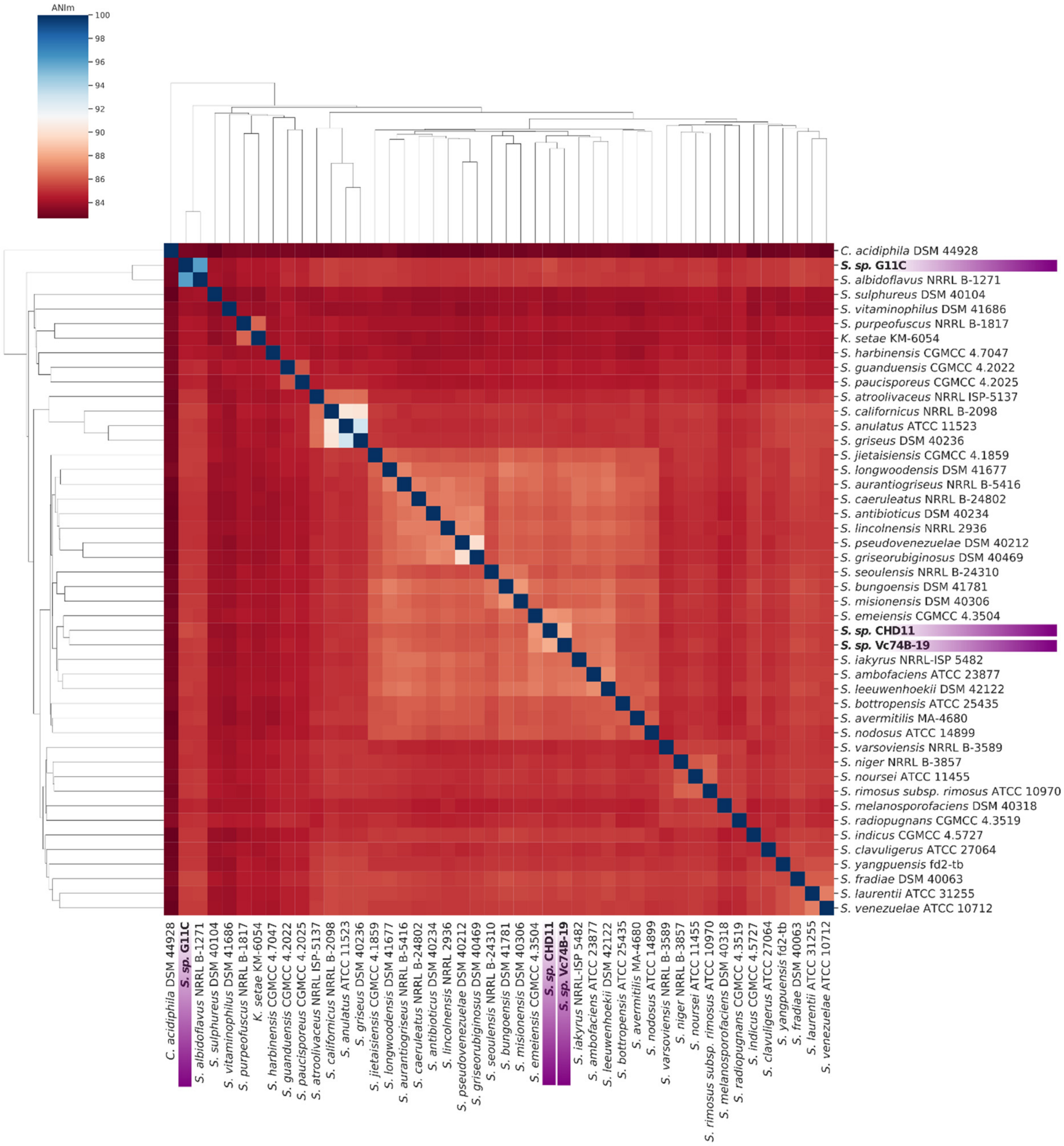

Figure S1. ANIm matrix of genomes employed for the phylogeny. Row and columns are sorted by automatic hierarchical clustering in the seaborn package. Color scale indicates ANIm values.

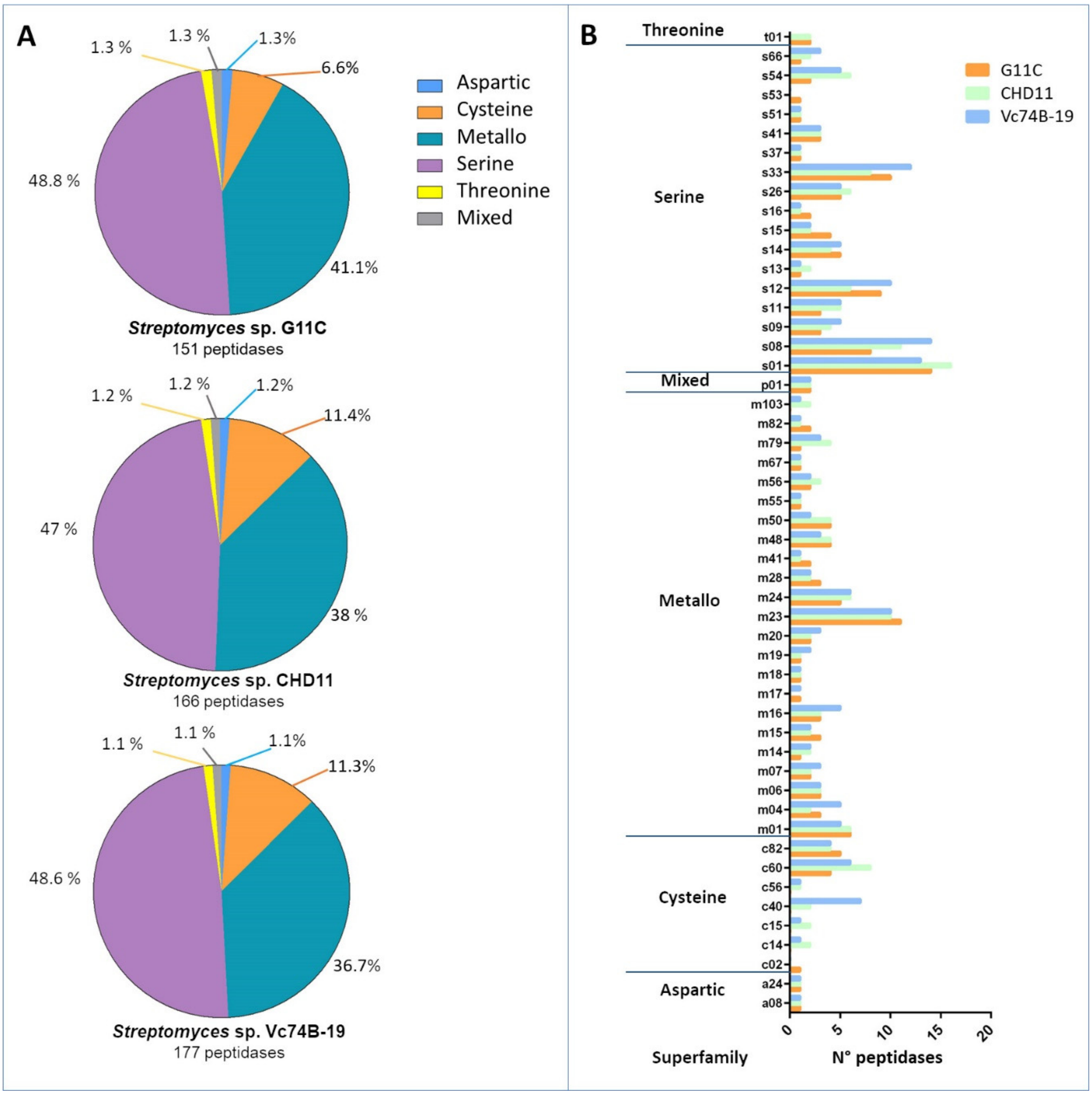

Figure S2. Classification of putative peptidases from Streptomyces strains G11C, CHD11 and Vc74B-19 using MEROPS database. (**A**) Relative abundance of peptidase super-families (aspartic, cysteine, metallo-, serine, threonine, and mixed peptidase super-families). Colours represent different peptidase super-families. (**B**) Abundance of peptidase families encountered in strain G11C, CHD11 and Vc74B-19 genomes. Colours represent putative peptidases belonging to different strains.

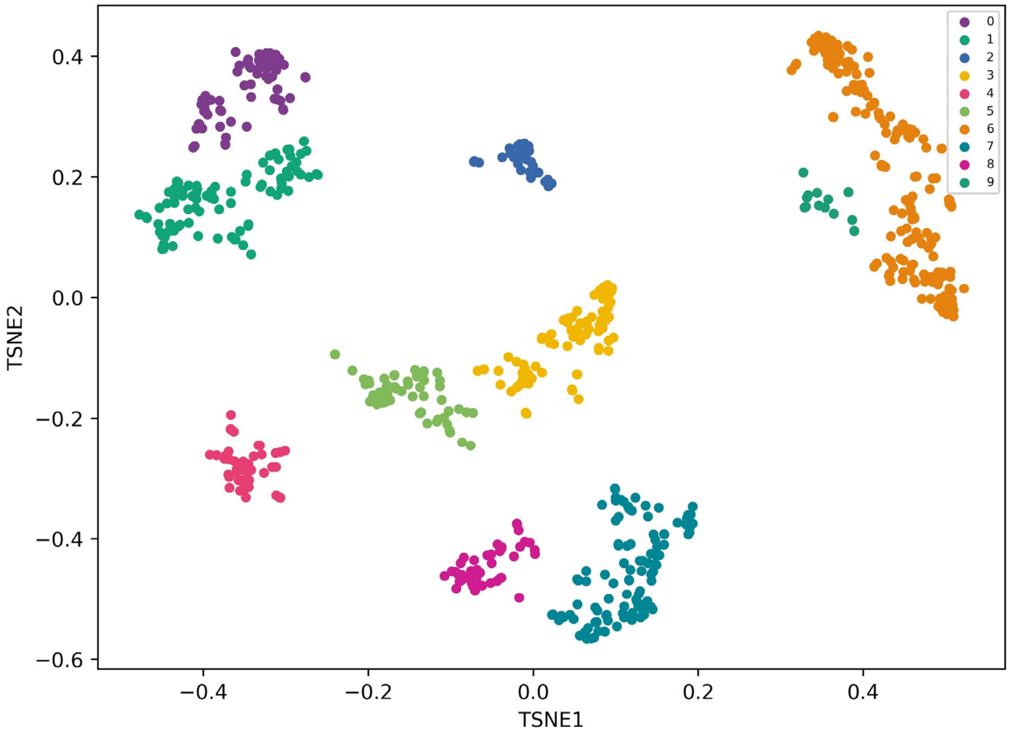

Figure S3. Clustering of t-SNE points using the DBSCAN algorithm. t-SNE group numbering is indicated in the legend.

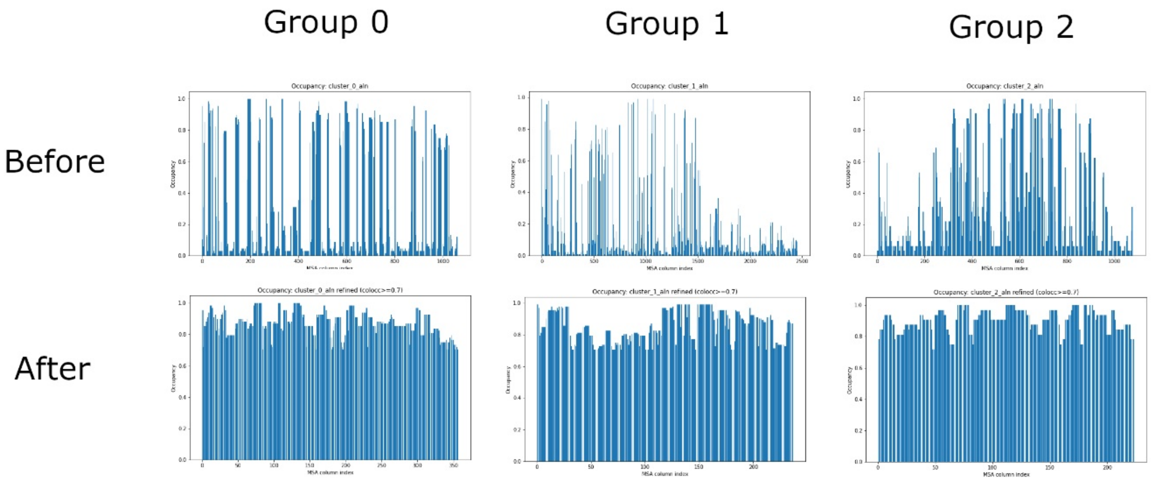

Figure S4. Occupancy plots. Occupancy plots (number of times a residue is found in a position across sequences) for the multiple sequence alignments (MSA) of the t-SNE group 0,1,2 before and after the position removal step by an occupancy threshold of 70%. For each group, the change in the number of positions for group 0 was 1059/356, for group 1 was 2454/236 and for group 2 was 1075/223, respectively.

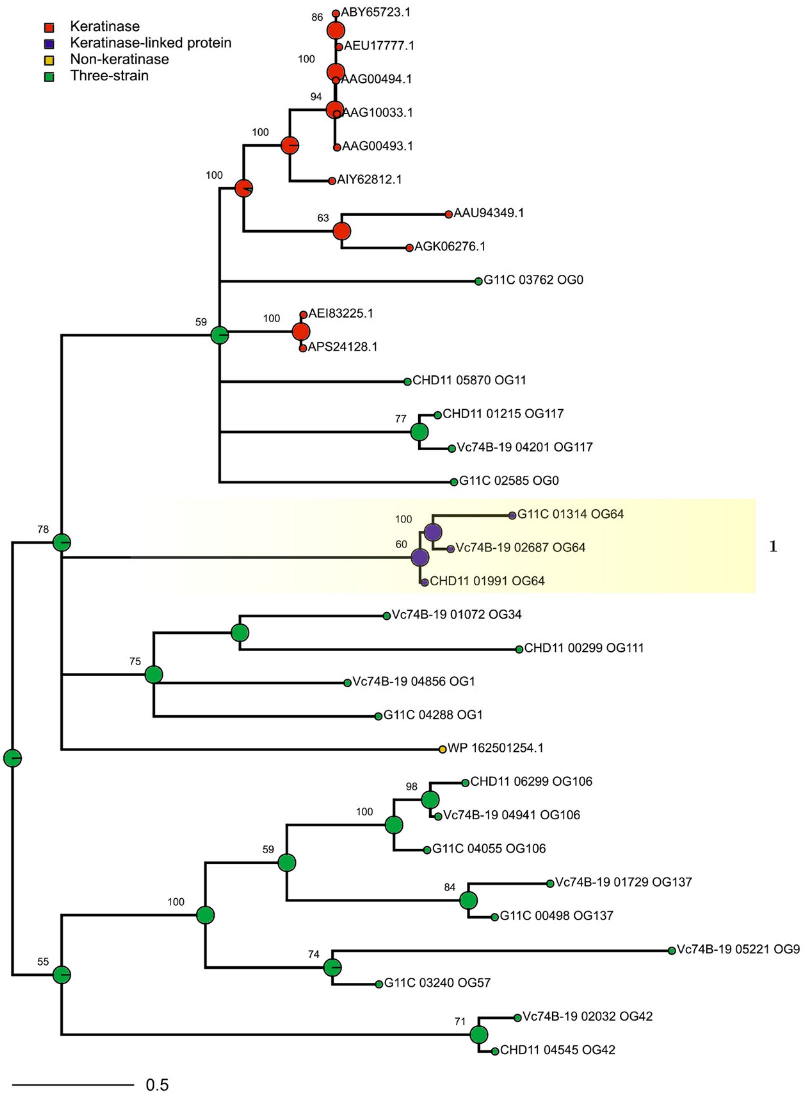

Figure S5. Maximum likelihood tree from the filtered MSA of t-SNE group 2 sequences. Ancestral state probabilities inferred in internal nodes for each category (functional keratinase, keratinase-linked protein, three-strain, and non-keratinase) are depicted as pie charts, with a total of 1 for each pie chart. Support values based on bootstrapping are indicated for each node. Selected clades under stipulated criteria are enclosed by light red boxes, while discarded clades are enclosed by light yellow boxes.

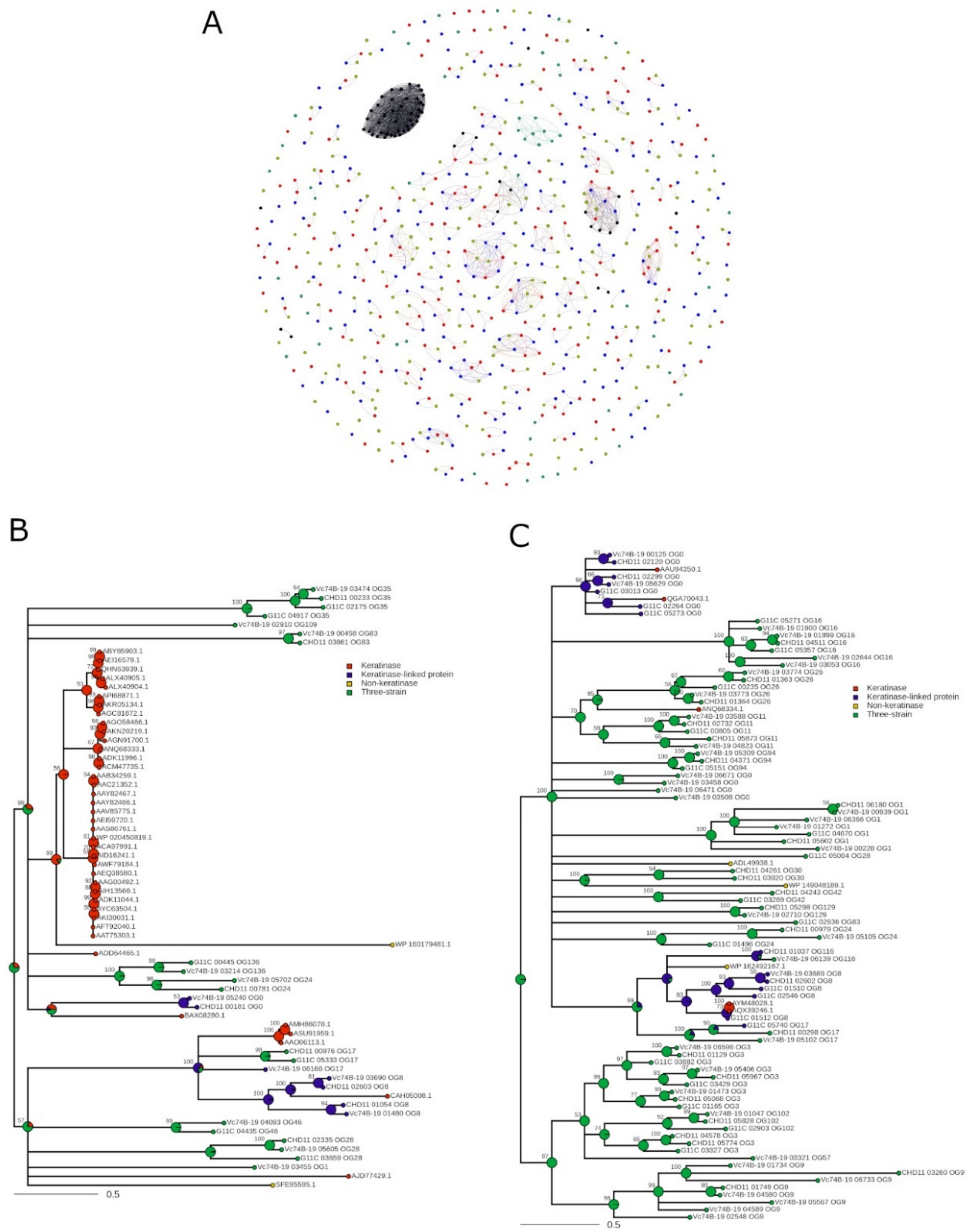

Figure S6. Protease similarity network using e-value threshold 1e-80 and Maximum likelihood trees from filtered MSA of t-SNE group 0 and t-SNE group 1 sequences. (**A**) Protease similarity network including the three-strain (584 sequences), keratinase (61 sequences), and non-keratinase (50 sequences) datasets. e-value threshold of the blast alignment for the network is 1e-80. Each node represents an identified putative protease and the colour fill indicates the origin of the sequence: red, strain G11C; green, strain CHD11; blue, strain Vc74B-19; black, functionally known keratinases; yellow, putative non-keratinases. Edge transparency was adjusted to represent e-value difference: darker edges correspond to smaller e-values. (**B**) & (**C**) Maximum likelihood trees from filtered MSA of t-SNE group 0 and t-SNE group 1 sequences. Ancestral state probabilities inferred in internal nodes for each category (functional keratinase, keratinase-linked protein, three-strain, and non-keratinase) are depicted as pie charts, using category mapping derived for the network with e-value cutoff 1e-80. Support values based on bootstrapping are indicated for each node.
