# Supplementary figures and images for: An Integrative Bioinformatic Analysis for Keratinase Detection in Marine-Derived Streptomyces
Source: Mar Drugs. 2021 May 21;19(6):286. doi: 10.3390/md19060286 (PMC8224001; doi:10.3390/md19060286)

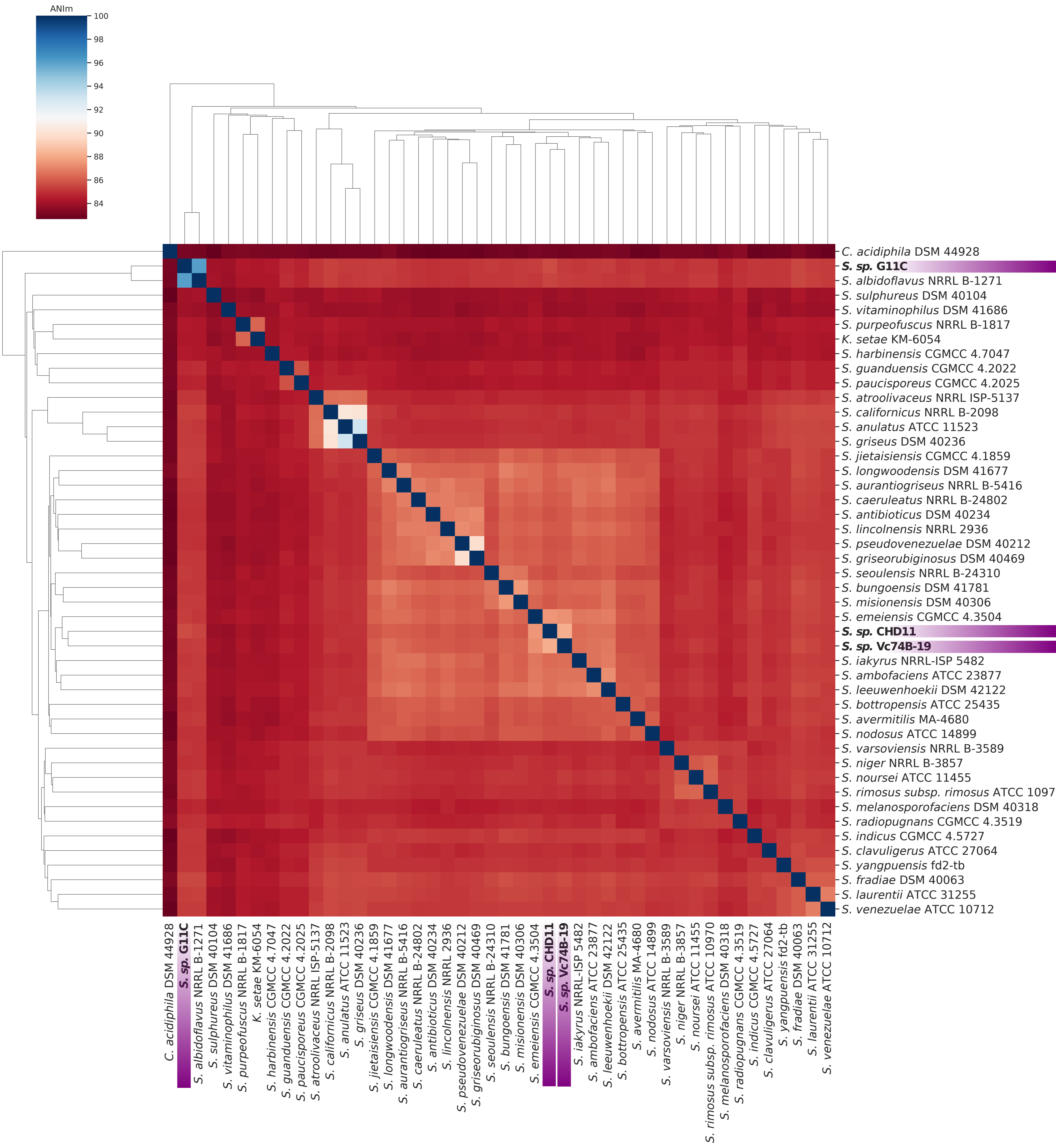

Supplement: Supplementary file 1 [file marinedrugs-19-00286-s001.zip › marinedrugs-1203633-SI/Supplementary_files5.13/Fig_S1_mod.pdf]

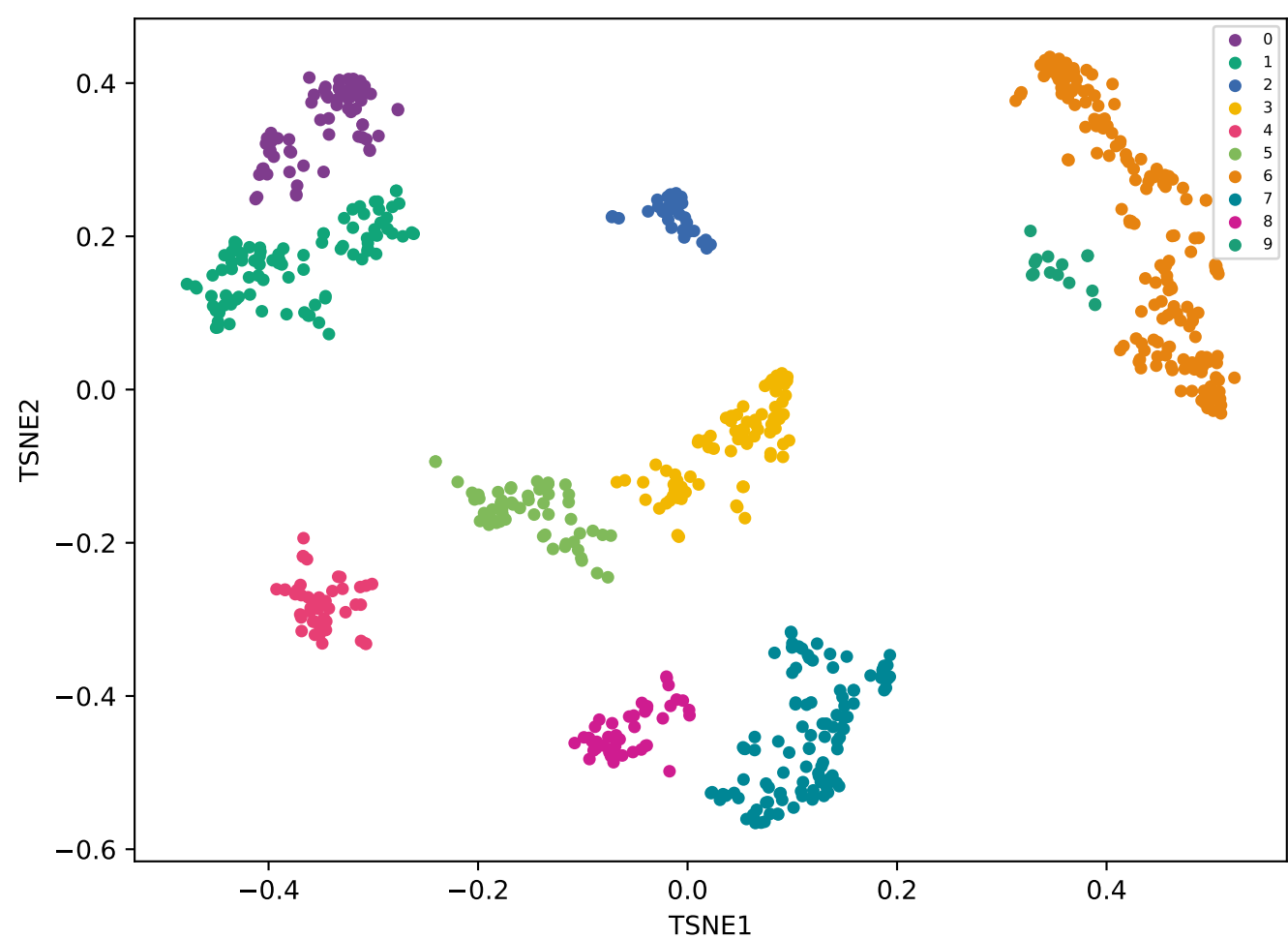

Supplement: Supplementary file 1 [file marinedrugs-19-00286-s001.zip › marinedrugs-1203633-SI/Supplementary_files5.13/Fig_S3.pdf]

# Group 0

# Group 1

# Group 2

Before

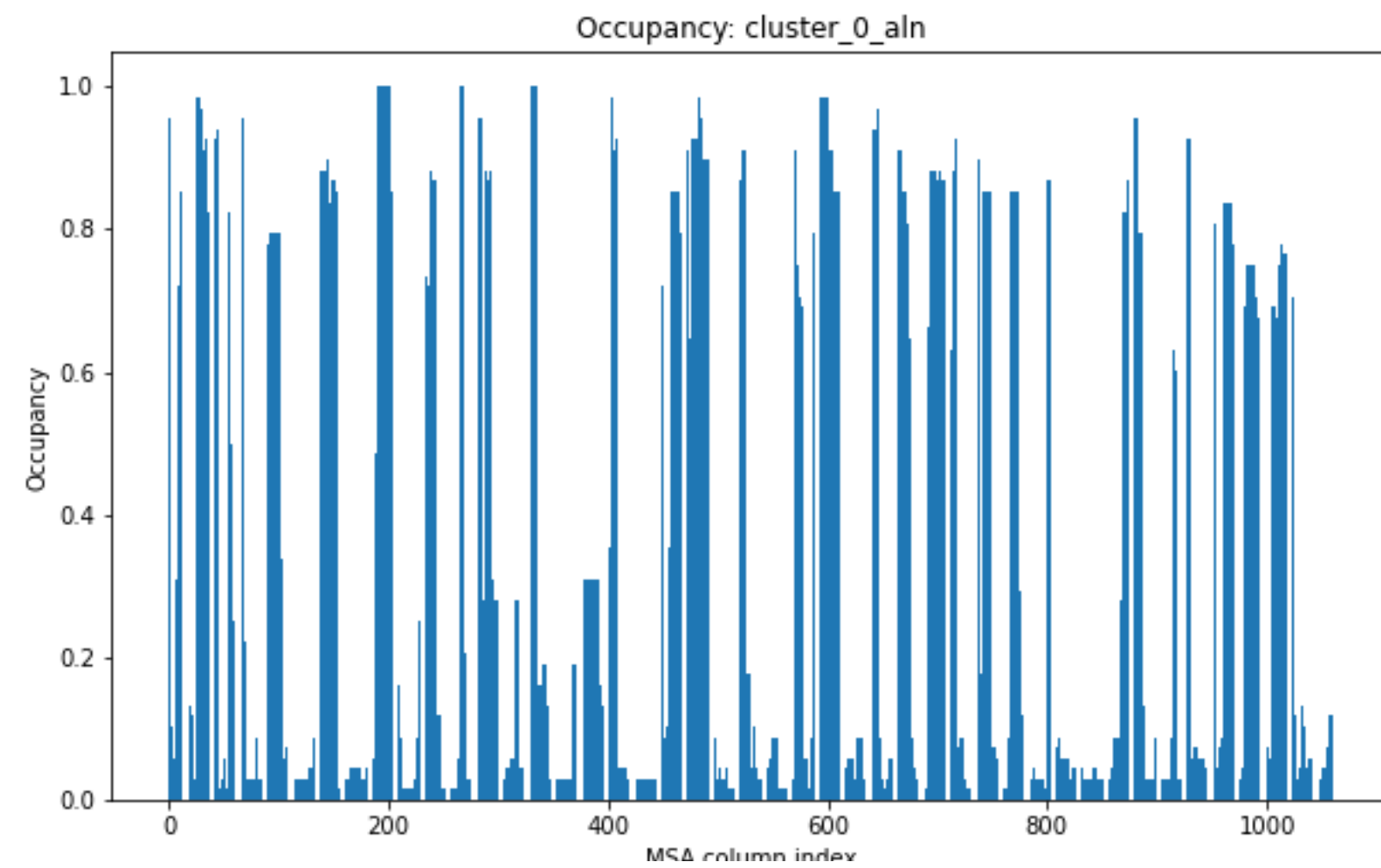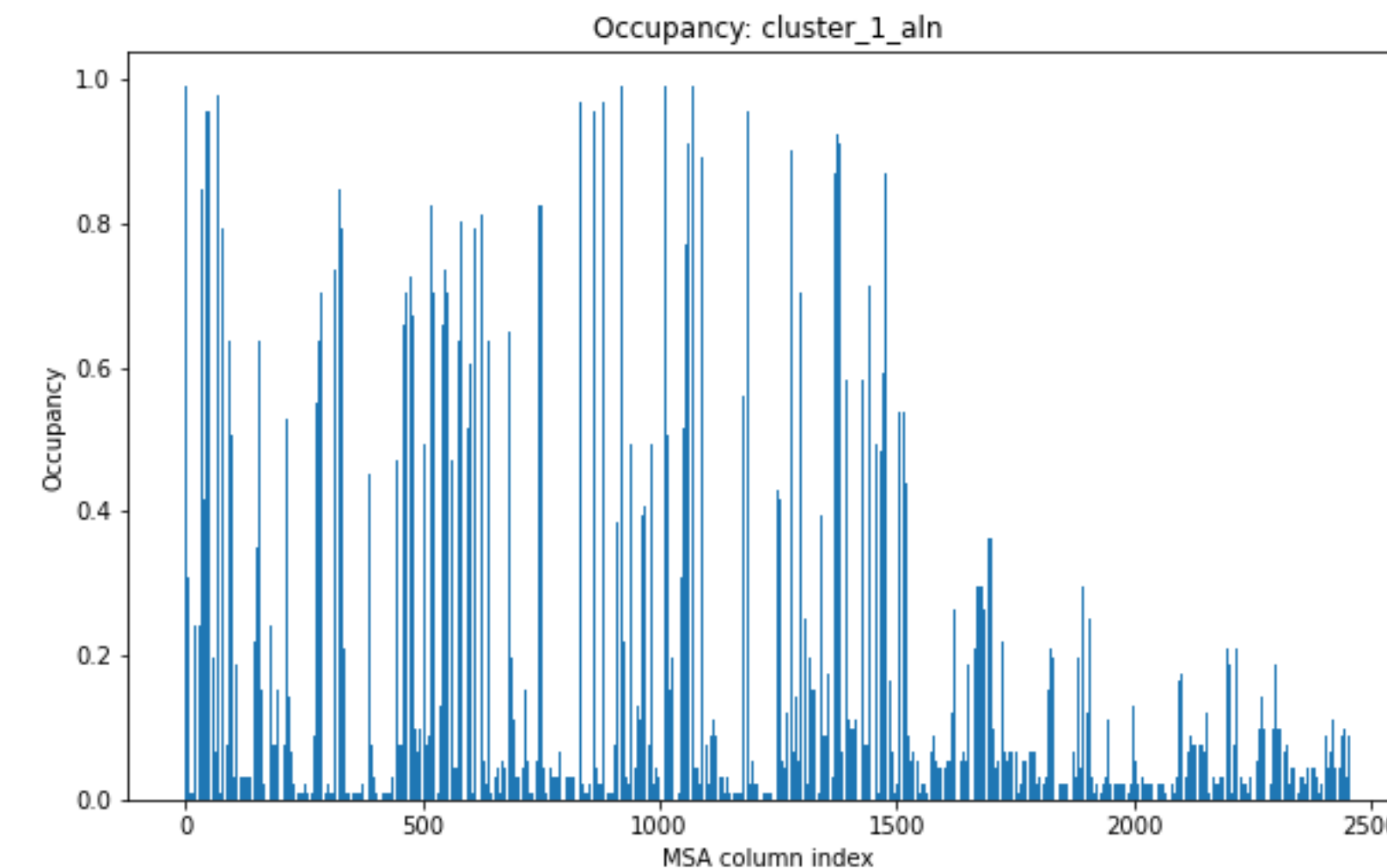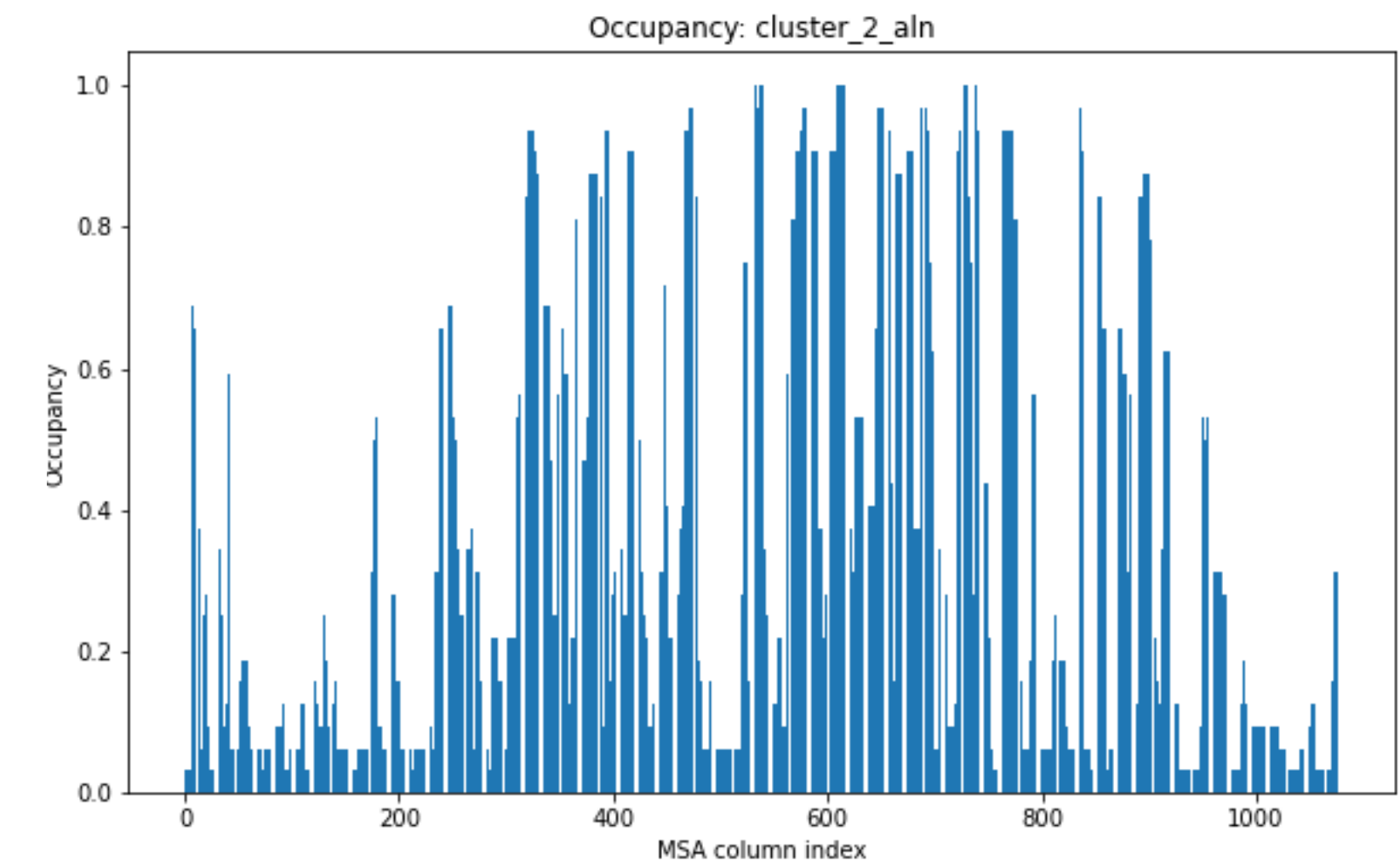

After

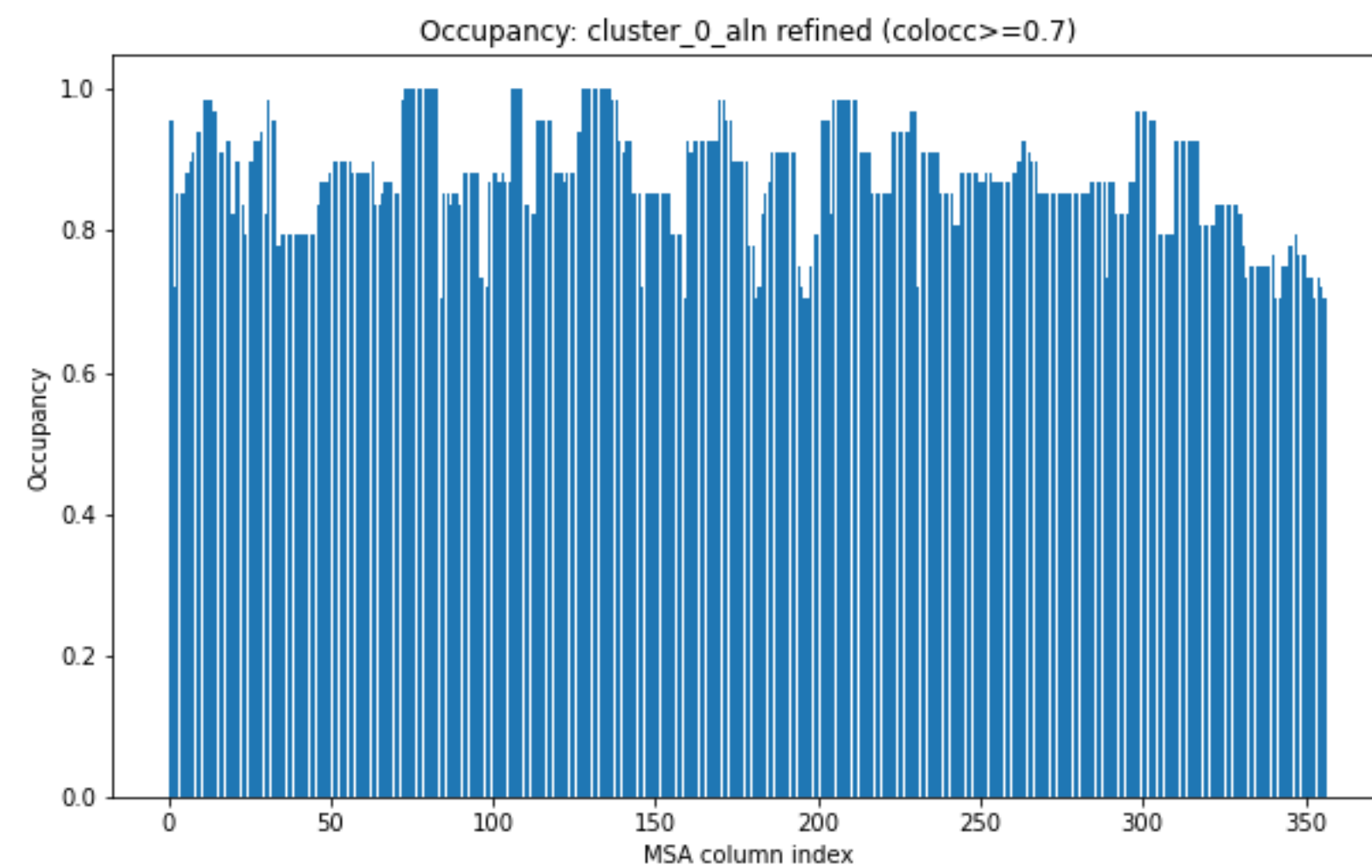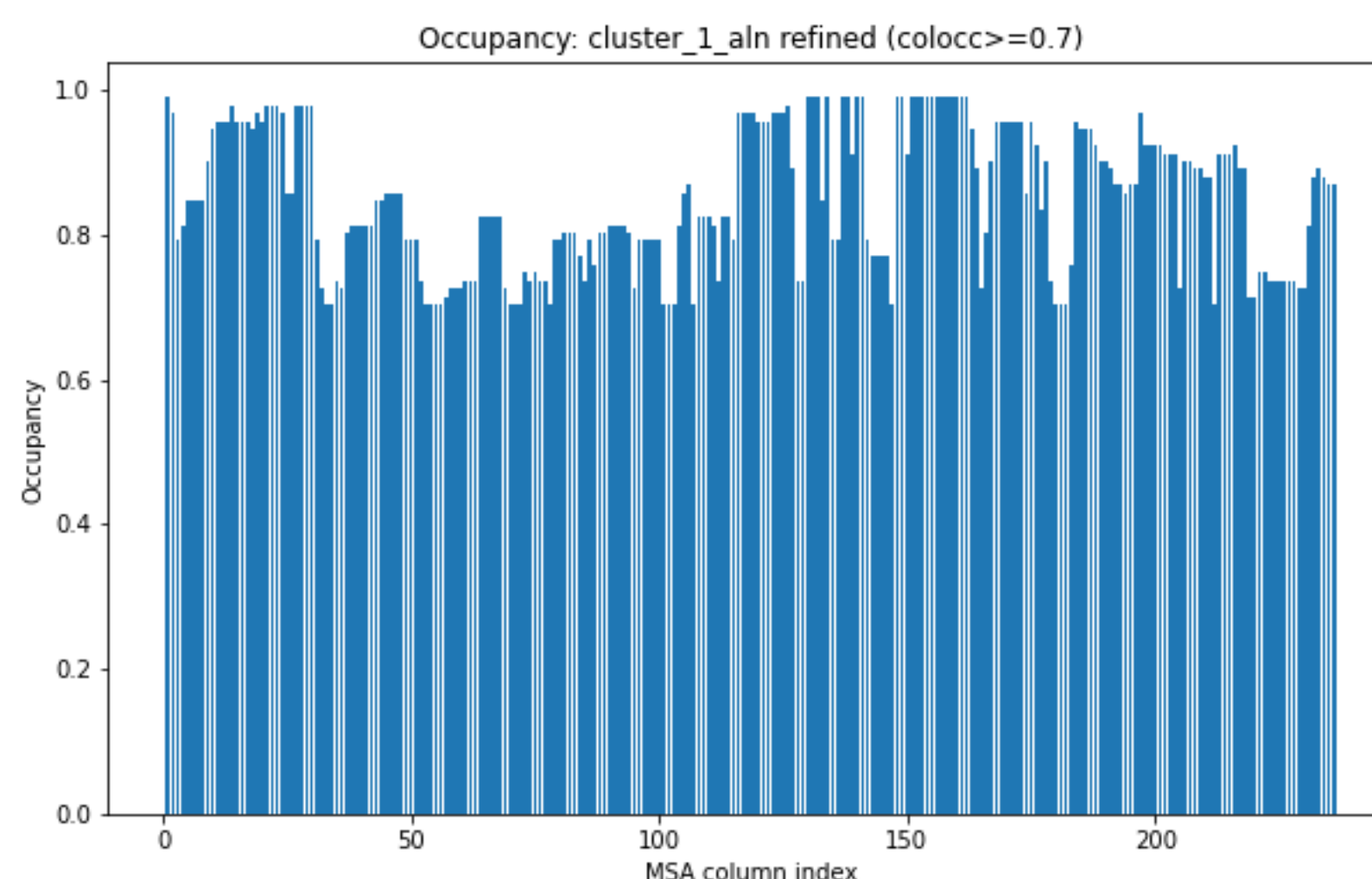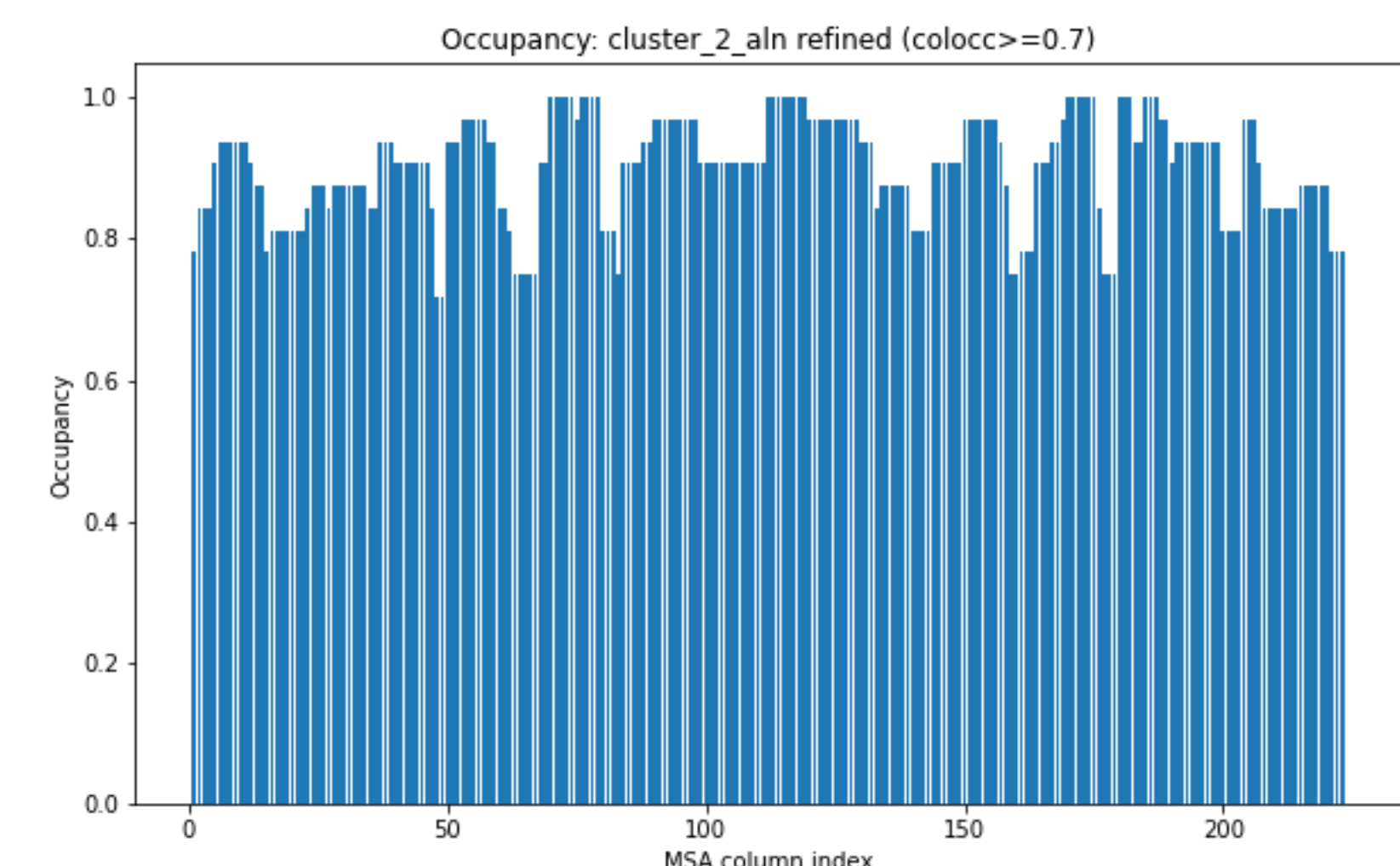

Supplement: Supplementary file 1 [file marinedrugs-19-00286-s001.zip › marinedrugs-1203633-SI/Supplementary_files5.13/Fig_S4.pdf]

- Keratinase
- Keratinase-linked protein
- Non-keratinase
- Three-strain

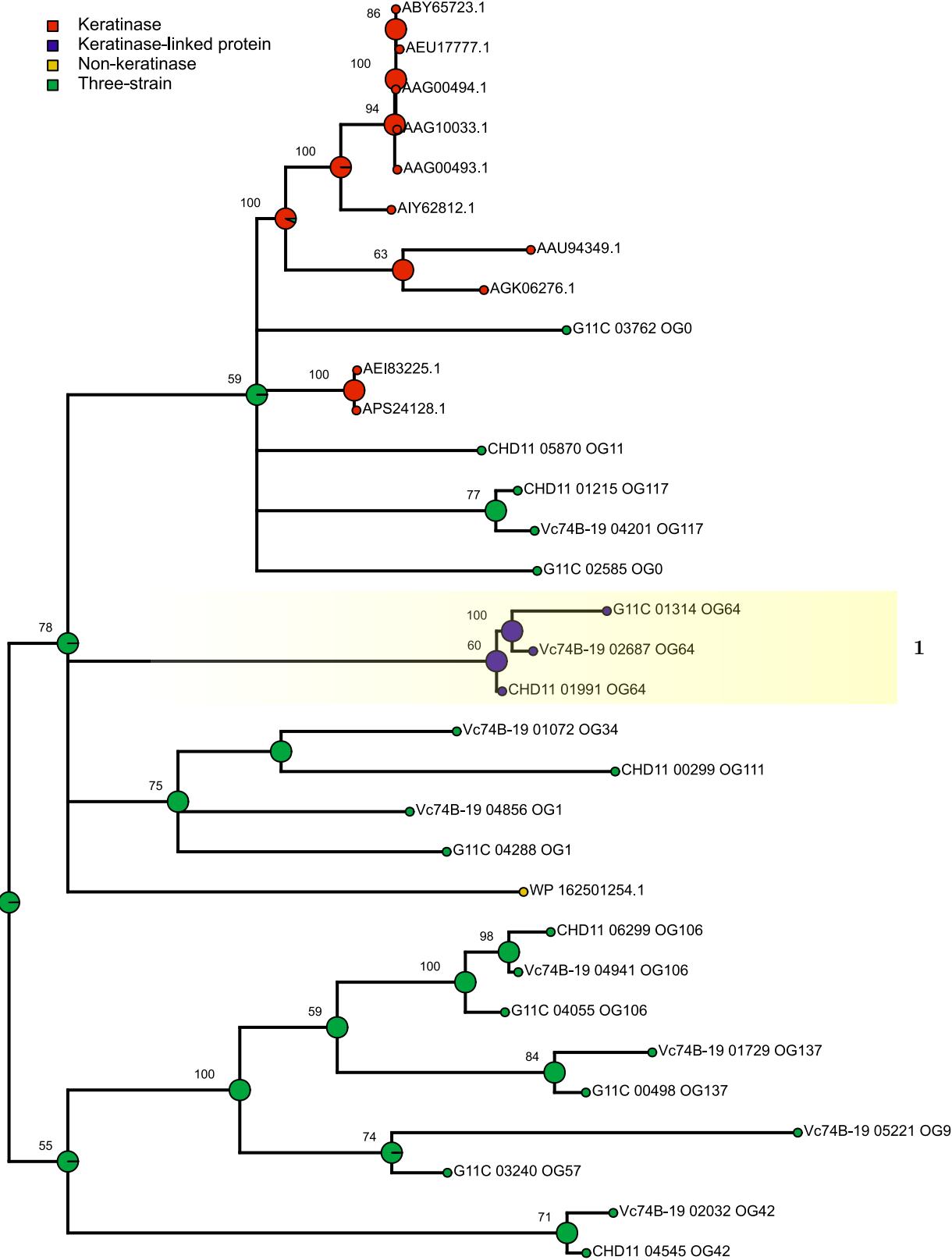

1

0.5

Supplement: Supplementary file 1 [file marinedrugs-19-00286-s001.zip › marinedrugs-1203633-SI/Supplementary_files5.13/Fig_S5.pdf]

A

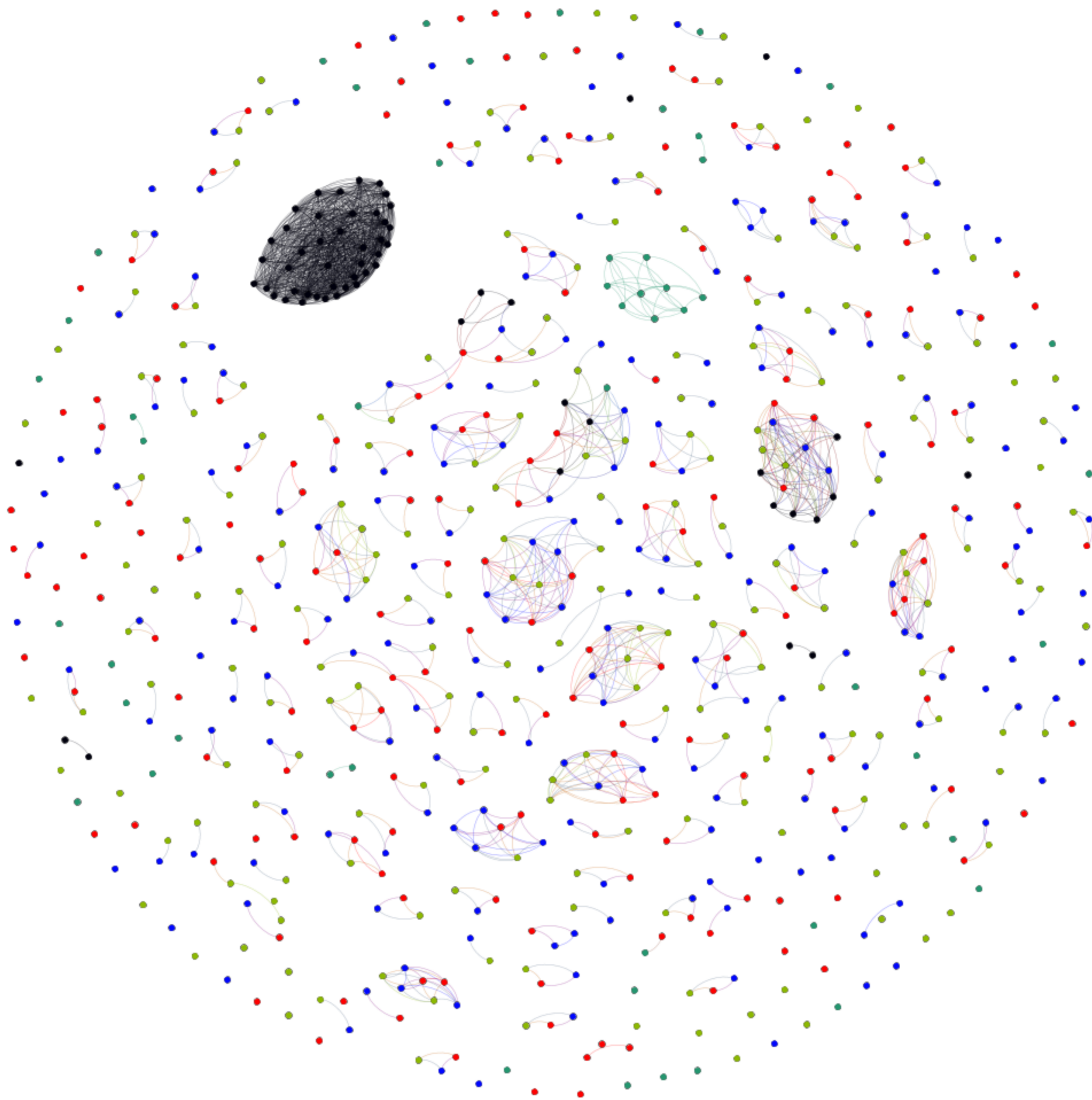

B

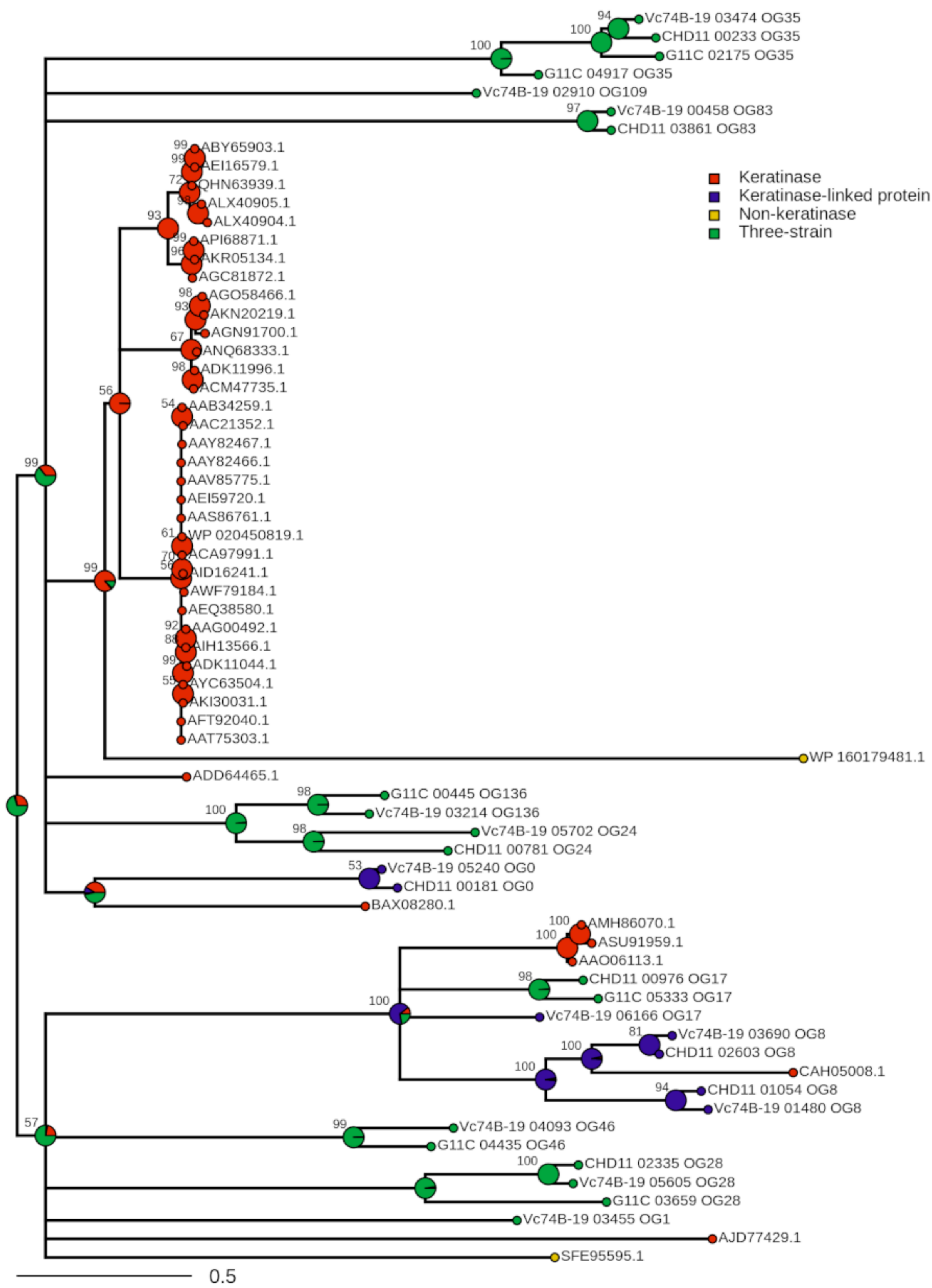

C

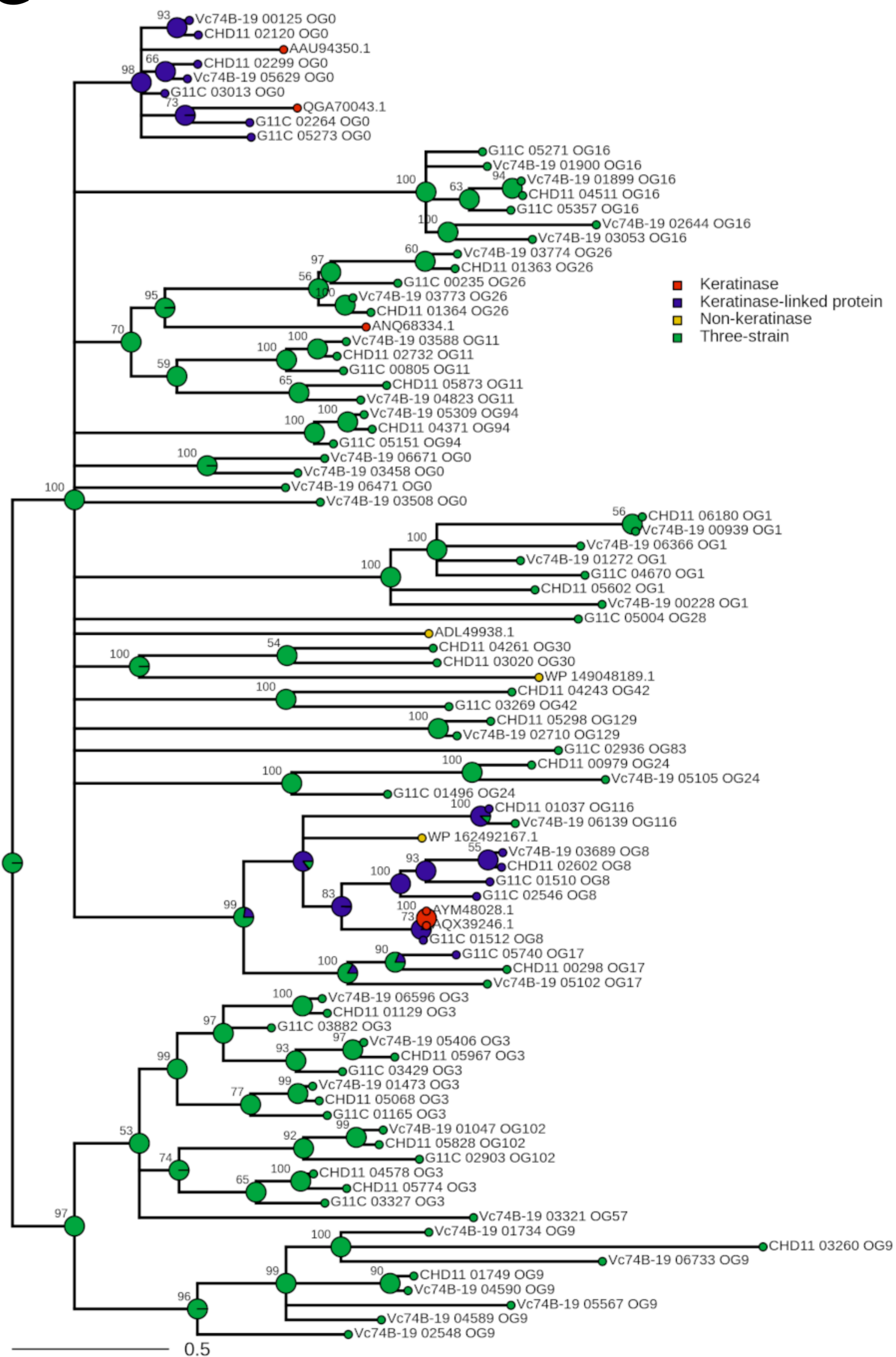

Supplement: Supplementary file 1 [file marinedrugs-19-00286-s001.zip › marinedrugs-1203633-SI/Supplementary_files5.13/Fig_S6.pdf]
